# Environmental Determinants of Post-Discharge Acute Respiratory Illness among Preterm Infants with Bronchopulmonary Dysplasia

**DOI:** 10.3390/ijerph21050648

**Published:** 2024-05-20

**Authors:** Jonathan J. Szeto, Joshua K. Radack, Sara B. DeMauro, Erik A. Jensen, Kathleen Gibbs, Nicolas P. Novick, Kristan A. Scott, Daria C. Murosko, Heather H. Burris, Timothy D. Nelin

**Affiliations:** 1 Perelman School of Medicine, University of Pennsylvania, Philadelphia, PA 19104, USA; jonathan.szeto@pennmedicine.upenn.edu; 2 Division of Neonatology, Department of Pediatrics, Children’s Hospital of Philadelphia, Philadelphia, PA 19104, USAdemauro@chop.edu (S.B.D.);; 3 Neonatal Follow-Up Program, Children’s Hospital of Philadelphia, Philadelphia, PA 19104, USA; 4 Newborn/Infant Chronic Lung Disease Program, Children’s Hospital of Philadelphia, Philadelphia, PA 19104, USA; 5 Leonard Davis Institute of Health Economics, Philadelphia, PA 19104, USA; 6 Center of Excellence in Environmental Toxicology, Perelman School of Medicine, University of Pennsylvania, Philadelphia, PA 19104, USA

**Keywords:** bronchopulmonary dysplasia, respiratory illness, environmental justice index

## Abstract

Objective: To analyze the association of components of the Centers for Disease Control and Prevention (CDC) Environmental Justice Index (EJI) with respiratory health outcomes among infants with bronchopulmonary dysplasia (BPD) within one year after discharge from the neonatal intensive care unit. Methods: This was a retrospective cohort study of a cohort of preterm infants with BPD. Multivariable logistic regression models estimated associations of EJI and its components with medically attended acute respiratory illness, defined as an ED visit or inpatient readmission, within one year of discharge from the neonatal intensive care unit. A mediation analysis was conducted to evaluate how environmental injustice may contribute to racial disparities in acute respiratory illness. Results: Greater EJI was associated with an increased risk of medically attended respiratory illness (per EJI standard deviation increment, aOR 1.38, 95% CI: 1.12–1.69). Of the index’s components, the Environmental Burden Module’s Air pollution domain had the greatest association (aOR 1.44, 95% CI: 1.44–2.61). With respect to individual indicators within the EJI, Diesel Particulate Matter (DSLPM) and Air Toxic Cancer Risk (ATCR) demonstrated the strongest relationship (aOR 2.06, 95% CI: 1.57–2.71 and aOR 2.10, 95% CI: 1.59–2.78, respectively). Among non-Hispanic Black infants, 63% experienced a medically attended acute respiratory illness as compared to 18% of non-Hispanic White infants. DSLPM mediated 39% of the Black–White disparity in medically attended acute respiratory illness (*p* = 0.004). Conclusions: Environmental exposures, particularly air pollution, are associated with post-discharge respiratory health outcomes among preterm infants with BPD after adjusting for clinical, demographic, and social vulnerability risk factors. Certain types of air pollutants, namely, DSLPM, are more greatly associated with acute respiratory illness. Environmental exposures may contribute to racial disparities in medically attended acute respiratory illness among infants with BPD.

## 1. Introduction

Advancements in prenatal and neonatal intensive care have markedly improved survival rates for extremely preterm infants [1]. However, with increasing preterm infant survival, the prevalence of bronchopulmonary dysplasia (BPD) has increased [2,3,4]. BPD is the most common chronic morbidity diagnosed in infants born extremely preterm [5,6]. The earlier in gestation infants are born, the more likely they are to develop BPD. For example, among infants born before 26 weeks gestation, 28–50% of survivors develop BPD compared to just 4% of infants born between 30 and 32 weeks gestation [5]. BPD results in structural changes in lung architecture and pulmonary vasculature that can lead to enduring impairments in gas exchange and cardiopulmonary function [3,7]. As a result, children with BPD experience increased healthcare utilization. Up to half of infants with BPD are readmitted to the hospital for respiratory illness within the first year after birth [8,9]. This may worsen infant and family quality of life and pose a significant financial burden to families [4,7].

Neighborhood social vulnerability, the cumulative negative effect caused by various exogenous, residential area-level stressors on human health, is associated with the risk of preterm birth [10]. As such, preterm infants are disproportionately discharged from neonatal intensive care units (NICUs) to socially vulnerable neighborhoods. Prior analyses by this group and others have shown that neighborhood social vulnerability is associated with medically attended respiratory illness and hospital readmissions among infants with BPD [9,11]. Socially vulnerable communities experience disproportionate exposure to environmental toxicants, which may drive disparities in health outcomes. For example, among infants with BPD, ambient and indoor air pollution adversely affect respiratory outcomes as indicated by the receipt of prescriptions for inhaled corticosteroids, use of systemic steroids, emergency department visits, and delayed weaning from supplemental oxygen [12,13,14].

We sought to investigate how various social and environmental factors may individually and collectively impact acute healthcare utilization for respiratory illness in preterm infants with BPD. We aimed to characterize the relationship of the Centers for Disease Control and Prevention (CDC) Environmental Justice Index (EJI) with medically attended acute respiratory illness. By using this multi-component index, our study builds upon existing literature by beginning to evaluate and quantify the differential impact of various social and environmental factors, notably, to directly compare four types of air pollutants and their association with acute respiratory illness among preterm infants with BPD. We speculated that exposure to greater environmental burden would be associated with an increased risk of medically attended acute respiratory illness in the first year after NICU discharge. To determine the extent to which differential exposure to air pollution may explain racial disparities in acute respiratory illness, we performed an exploratory mediation analysis.

## 2. Methods

### 2.1. Study Population

Preterm infants with BPD comprised the study cohort. Infants were included in this study if they were born <32 weeks’ gestation between 2010 and 2020, diagnosed with BPD according to the 2019 Neonatal Research Network Criteria, and survived to hospital discharge [15]. Infants who do not require supplemental respiratory support at 36 weeks’ post-menstrual age are not diagnosed with BPD and were excluded from the study. This yielded a patient cohort of 378 infants. Infant information was retrospectively obtained from the Children’s Hospital of Philadelphia (CHOP) electronic health record and a local research registry comprised of infants who received care within the CHOP Care Network and the University of Pennsylvania Health System. Infants were also given one of three grades based on their disease severity and mode of respiratory support used at 36 weeks’ PMA as defined by the 2019 Neonatal Research Network BPD definition. Grade 1 is defined as treatment with nasal cannula at flows ≤ 2 L/min. Grade 2 is defined as treatment with high-flow nasal cannula (>2 L/min) or non-invasive positive airway pressure. Grade 3 is defined as treatment with any form of invasive mechanical ventilation.

Infants missing documentation of home address at time of hospital discharge and those discharged to an address outside of the Philadelphia metropolitan region were excluded to decrease the likelihood of missing healthcare visits that occurred outside of the CHOP hospital network. For reference, there are three free-standing children’s hospitals that serve the metropolitan Philadelphia region and CHOP serves as the inpatient and post-discharge medical home for most infants with high-grade BPD in the region.

### 2.2. Exposures

The CDC EJI is composed of 3 modules reported for each census tract: the social vulnerability module (SVM); the environmental burden module (EBM); and the health vulnerability module (HVM). These modules are comprised of one to five domains, with each domain including one to seven indicators. In total, the EJI is comprised of 3 modules, 10 domains, and 36 indicators. The EJI index, as well as all variables in the SVM and EBM, has continuous variables with values ranging from 0 to 1. The granular air pollution measurement variables within the EBM were also derived at the census tract level. For example, the PM_2.5_ indicator was created by generating the mean annual percent of days with a daily average PM_2.5_ concentration over the National Ambient Air Quality Standard in each census tract, sorting the census tracts, and assigning a percentile ranking. For HVM variables, census tracts are given a value of 0.2, 0.4, 0.6, 0.8, or 1.0 based on survey data collected as part of the CDC’s Behavioral Risk Factor Surveillance System [16]. For the EJI composite, module, domain, and indicator rankings, the percentile represents the proportion of census tracts that are equal to or lower than the tract of interest in environmental burden. For instance, if the ranking of the EJI is 0.7, this means that 70% of census tracts in the United States cumulatively experience less severe environmental burden, and 30% of census tracts in the United States experience more severe cumulative environmental burden than that tract [16].

Our primary exposures of interest were census tract CDC EJI, EBM, and the components of the air pollution domain of EBM. The air pollution indicators are ozone, PM_2.5_, diesel particulate matter (DSLPM), and air toxic cancer risk (ATCR) [15]. Long-term exposure to ozone has been associated with an increased risk of respiratory mortality and a greater decline in lung function [17]. PM_2.5_ is comprised of aerosol particles smaller than approximately 2.5 μm in diameter and was responsible for an estimated 4.2 million annual premature deaths globally in 2015 [18]. DSLPM contains soot particles, metals, and metal oxides, which are both mutagenic and carcinogenic [19]. ATCR is a composite metric that estimates the risk of cancer based on the level of exposure to 140 different hazardous air pollutants, such as benzene, dioxin, formaldehyde, and ethylene oxide [16].

### 2.3. Outcomes

The primary composite outcome was any medically attended acute respiratory illness during the 12-month period after NICU discharge. This outcome was defined as either an ED visit or an inpatient readmission for acute respiratory illness identified using ICD 9 and 10 codes. Secondary outcomes were the individual components of the composite outcome: ED visits alone and inpatient readmission for acute respiratory illness. Inpatient readmissions were considered any readmission after initial discharge from the NICU for acute respiratory illness. Infants readmitted through an ED visit were included only in the readmission category and excluded from the ED visit category. Infants with separate and distinct episodes of ED visits and hospital admissions were similarly included only in the hospital admission category and excluded from the ED category. Episodes were considered distinct if they were separated by >24 h. Patients who visited the ED or were readmitted more than once were counted only once in this study.

### 2.4. Statistical Analysis

Student’s two-sided *t* test, Wilcoxon rank sum tests, and Chi-square tests were used to perform bivariable analyses describing unadjusted associations of infant characteristics and environmental exposures with medically attended acute respiratory illness.

We characterized associations of one standard deviation (SD)-unit increment higher EJI, as well as each of the various modules, domains, and indicators that comprise the EJI, with medically attended acute respiratory illness using multiple logistic regression models. We adjusted for clinical and patient characteristics, identified *a priori*, that prior studies have shown to be associated with readmission such as infant sex and BPD grade, as well as those that might confound associations of environmental exposures with ED visits and readmissions: gestational age at birth; birth year; and insurance status (private vs. public). Additionally, in models assessing air pollution exposures, we also adjusted for census tract neighborhood deprivation quantified using the Brokamp Deprivation Index since community socioeconomic status can act as both a confounder and an effect modifier between air pollution and adverse health outcomes [20,21]. Notably, in the models with EJI as the exposure, we did not adjust for deprivation as similar indicators are already included in the EJI. Analyses were repeated using multinomial logistic regression to disaggregate the composite outcomes of medically attended acute respiratory illness and quantify associations of EJI, EBM, and the components of the air pollution domain of EBM with the risk of having an ED visit alone or at least one inpatient readmission. Finally, in a separate model, we additionally adjusted for race and ethnicity to observe whether associations persisted with this adjustment. Infants were placed into mutually exclusive categories of non-Hispanic Black, non-Hispanic White, and a third group of all other races and ethnicities due to small sample sizes. Category assignment was based on information from the electronic health record.

A causal mediation analysis was also performed to quantify the disproportionate impact that high levels of environmental burdens may have on respiratory health among non-Hispanic Black compared to non-Hispanic White infants. Our analysis for this subcohort of patients was performed following VanderWeele’s approach to causal mediation [22]. VanderWeele’s approach utilizes multivariable logistic regression models to quantify the direct association of an independent variable with a dependent variable and the indirect association of an independent variable with a dependent variable through higher levels of the mediator variables. In our analysis, the primary independent variable of interest was non-Hispanic Black race and ethnicity, the dependent variable was medically attended acute respiratory illness, and the mediator variables were EJI and its various modules, domains, and indicators. The multivariable logistic regression model used in the mediation analysis was adjusted for gestational age, infant sex, birth year, BPD grade, insurance status, and neighborhood deprivation as potential cofounders. No adjustments for multiple comparisons were made in this exploratory analysis.

The Institutional Review Board (IRB) of CHOP (IRB 20-018358) approved this study. R statistical software (v4.0.2; R Core Team 2021) was used to perform all analyses.

## 3. Results

**Figure 1:**
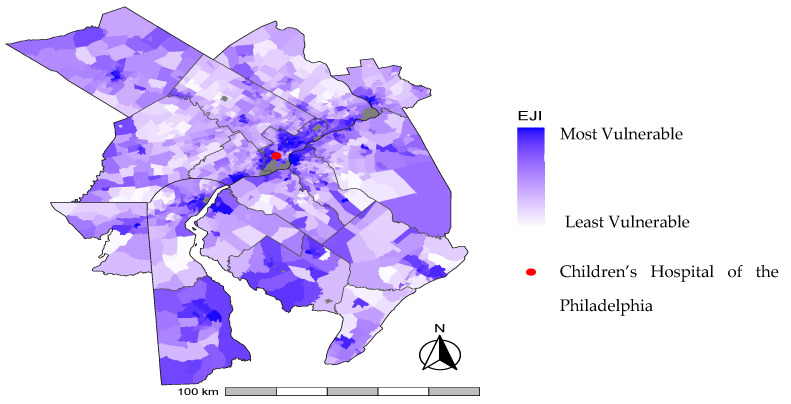
Environmental Justice Index (EJI) across metropolitan Philadelphia. Depicts EJI by census tract across the study area as calculated by the Centers for Disease Control and Prevention (CDC). Data Source: Centers for Disease Control and Prevention (CDC ATSDR and CDC NCEH, 2022). Map generated using ggplot2 package [23] with R software (v4.3.2).

**Figure 2:**
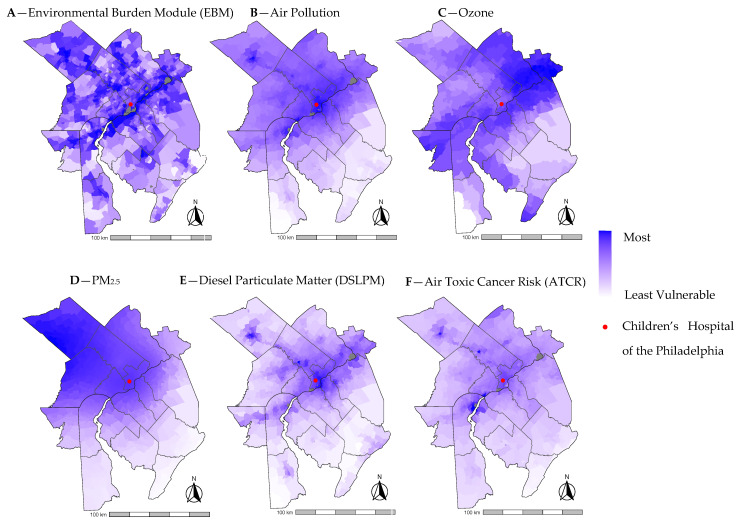
Environmental Justice Environmental Burden indices by census tract for metropolitan Philadelphia: (**A**) Depicts aggregate environmental burden by census tract. (**B**) Depicts aggregate air pollution index of the four pollutant indicators by census tract. (**C**) Depicts ozone vulnerability by census tract. (**D**) Depicts PM_2.5_ vulnerability by census tract. (**E**) Depicts DSLPM vulnerability by census tract. (**F**) Depicts ATCR vulnerability by census tract. Data Source: Centers for Disease Control and Prevention [16]. Maps generated using ggplot2 package [23] with R software (v4.3.2).

We identified 378 infants in this cohort with BPD at 36 weeks’ PMA. The mean (SD) EJI of the cohort was 0.69 (0.27) (Table 1). Figure 1 displays variation in census tract EJI across the metropolitan Philadelphia region. The mean (SD) EBM and air pollution of the cohort were 0.79 (0.15) and 0.82 (0.14), respectively. With respect to individual indicators within air pollution, mean (SD) ozone, PM_2.5_, DSLPM, and ATCR of the cohort were 0.81 (0.08), 0.79 (0.14), 0.72 (0.16), and 0.62 (0.16), respectively (Table 1). Figure 2 displays variations in census tract EBM, air pollution, and the four air pollution indicators across the study area. In bivariate analyses, EJI, SVM, HVM, Air pollution, PM_2.5_, DSLPM, ATCR, neighborhood deprivation, and infant race and ethnicity were all associated with medically attended acute respiratory illness in the first year after NICU discharge (All *p* < 0.05) (Table 1).

### 3.1. EJI and Medically Attended Acute Respiratory Illness

In unadjusted models, we detected an association of higher EJI (OR 1.38, 95% CI: 1.12–1.69), air pollution domain (OR 1.94, 95% CI: 1.44–2.61), PM_2.5_ indicator (OR 1.72, 95% CI: 1.27–2.32), DSLPM indicator (OR 2.06, 95% CI: 1.57–2.71), and ATCR indicator (OR 2.10, 95% CI: 1.59–2.78) with the composite medically attended acute respiratory illness for a one SD increment of increase in each of these indices (Table 2). There were also significant associations of these indicators with ED visits alone and inpatient readmissions alone in the first year after NICU discharge.

With multivariable adjustment for infant sex, BPD grade, gestational age at birth, birth year, insurance status (private vs. public), and neighborhood deprivation (except in the EJI model), associations remained significant for EJI, air pollution domain, PM_2.5_ indicator, DSLPM, and ATCR with the composite outcome of medically attended acute respiratory illness (Table 2). EJI, DSLPM, and ATCR also remained associated with ED visits while EJI, air pollution domain, PM_2.5_ indicator, DSLPM, and ATCR remained associated with inpatient readmissions. There was no statistically significant association between acute respiratory illness and either ozone or EBM in any of the models. With further adjustment for race and ethnicity, effect estimates were similar with significant associations persisting for the air pollution domain, DSLPM, and ATCR with the composite outcome (Table S1, Supplementary Materials).

### 3.2. Mediation Analysis to Quantify the Contribution of EJI to Racial Disparities

**Figure 3:**
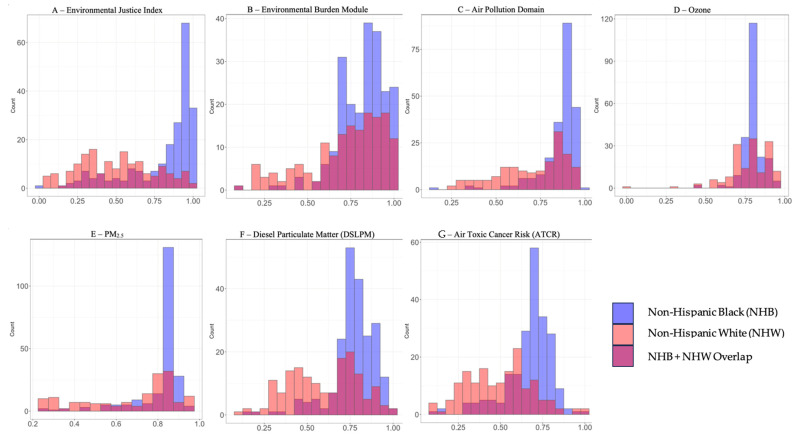
Distribution of index values of primary independent variables of interest among metropolitan Philadelphia patient cohort disaggregated by race: Non-Hispanic Black, Non-Hispanic White, and overlap between the Non-Hispanic Black and Non-Hispanic groups. Histograms depict the number of patients in the cohort disaggregated by race per 0.05 increment for each primary independent variable of interest: (**A**) is a histogram of EJI distribution. (**B**) is a histogram of EBM distribution. (**C**) is a histogram of air pollution distribution. (**D**) is a histogram of ozone distribution. (**E**) is a histogram of PM_2.5_ distribution. (**F**) is a histogram of DSLPM distribution. (**G**) is a histogram of ATCR distribution. Plots generated using ggplot2 package [23] with R software (v4.3.2).

Non-Hispanic Black infants were more likely than non-Hispanic White infants to have a medically attended acute respiratory illness (58% vs. 32%, *p* < 0.001), ED visit (30% vs. 6%, *p* < 0.001), and inpatient readmissions (42% vs. 26%, *p* < 0.001). Figure 3 displays the distribution of all seven environmental exposures of interest among the subset of non-Hispanic Black (*n* = 189) and non-Hispanic White (*n* = 99) infants. Air pollution, DSLPM, and ATCR had significant mediation effects on the composite outcome of medically attended acute respiratory illness as well as ED visits alone and inpatient readmissions (Table 3). DSLPM mediated 39% of the disparity in the composite outcome of medically attended acute respiratory illness (*p* = 0.004), 40% of the disparity in ED visits (*p* = 0.008), and 40% of the disparity in inpatient readmissions (*p* = 0.002). We did not detect statistically significant mediation of the racial disparity in medically attended acute respiratory illness by EJI, EBM, Ozone, or PM_2.5_ in this patient cohort.

## 4. Discussion

In a contemporary cohort of preterm infants with BPD discharged from a single hospital system, we found that higher census tract EJI was associated with increased odds of respiratory illness that resulted in medically attended acute respiratory illness, ED visits, and inpatient readmissions in the first year after NICU discharge, thus confirming our original hypothesis. When disaggregated into the components of the EJI, we found that while the composite environmental burden module was not associated with medically attended acute respiratory illness, PM_2.5_, DSLPM, and ATCR were all associated with medically attended acute respiratory illness.

With the exception of ATCR, metropolitan Philadelphia is in the top quartile of air pollution exposure in the entire United States based on the CDC EJI [16]. This analysis showed that there are substantial racial disparities with respect to census tract level EJI, EBM, and air pollution exposures as non-Hispanic Black individuals were more likely to reside in census tracts with higher index scores across all EJI variables compared to non-Hispanic White individuals. Similarly, within the cohort, there was a statistically significant racial disparity in acute respiratory illness. Our findings support the hypothesis that a portion of the disparity in outcomes may be explained by higher exposure to air pollution among non-Hispanic Black individuals compared to non-Hispanic White individuals, notably by DSLPM and ATCR. As such, this study further demonstrates the potential role of the physical environmental and environmental toxicants, such as air pollution, in health inequities.

To our knowledge, this is the first study to look at the CDC EJI, which combines social vulnerability, environmental burden, and health vulnerability factors, in the context of infants with BPD. The EBM’s lack of association with medically attended acute respiratory illness suggests that not all environmental factors may be associated with an increased risk of readmission in infants with BPD. Indicators related to water pollution, hazardous sites, the built environment, and transportation infrastructure are included in the EBM. These indicators may pose other health risks for preterm infants, but inhaled toxicants—as measured via air pollution—have face validity and evidence demonstrating a link to respiratory illness [24]. The significant association of aggregate air pollution and three of the four air pollution indicators with acute respiratory illness suggests that these specific physical environmental variables may be important risk factors for acute respiratory illness in patients with BPD. These findings are consistent with a prior study of 800 infants in Baltimore and Philadelphia that showed that ED visits in BPD patients had a 78% greater association with higher air pollution exposure at the county level as compared to lower air pollution [15]. Our study confirms and further expands upon these findings by using a more granular geographic exposure area at the census tract level. Census tracts are substantially smaller than counties; in 2020, Philadelphia County was subdivided into 408 distinct census tracts [25]. We also analyzed specific air pollution exposures to identify which factors might be more strongly associated with acute respiratory illness post-NICU discharge among infants with BPD. DSLPM had the strongest association with readmissions among infants with BPD. Importantly, roadways are a major source of DSLPM and Black individuals are more likely to live near large roadways in the United States [25,26]. Higher DSLPM can increase the production of inflammatory cytokines, which may explain its high association with acute respiratory illness and readmissions in the cohort [26].

The distribution of EJI values in our cohort is in line with past studies that have analyzed environmental inequities within the United States. Within our cohort, non-Hispanic Black infants tended to reside in areas with higher exposure to air pollution, which mirrors past studies investigating the effects of pollution on communities of color [27]. Our study expands upon these past results by calculating the potential contributions these exposures may have to observed racial disparities in outcomes. Redlining, a discriminatory practice of refusing financial services to individuals living in certain neighborhoods that have significant numbers of racial and ethnic minorities, has shaped the framework of American cities [28]. Historically, redlining is associated with closer proximity to fossil fuel powerplants among socioeconomically disadvantaged communities and communities of color, which demonstrates that community location as well as past and present racist policies could be an underlying cause of inequities in air pollution exposure, which may disproportionately contribute to acute respiratory illness in non-Hispanic Black infants with BPD [28,29].

The strengths of our study include an ample sample size over a 10-year period in a medically complex group of infants and the use of a pragmatic, evidence-based BPD grading scale [15] to create our cohort. The use of the mediation analysis allowed quantification of the potential contribution of various environmental factors on racial disparities in health outcomes among infants with BPD. Potential ascertainment or selection bias are limitations of this study as the cohort is only derived from infants admitted within the CHOP network and previously admitted to the NICU. However, many infants receive acute care at CHOP after initial hospital discharge due to the institute’s multidisciplinary care model and the fact that few other inpatient services exist in the region for infants with BPD [30]. The restriction of our study population to one metropolitan area and to a single health system reduces the generalizability of our findings to populations out of metropolitan Philadelphia and in different health systems as it was theoretically possible that infants could seek care at any of the three hospitals in the metro Philadelphia region. However, 365 (97%) of the 378 included infants had follow-ups in the CHOP network, implying high network participation in our study that likely extends to acute care episodes. Lastly, we did not have access to data on household or indoor air pollution exposures, such as environmental tobacco smoke, maternal environmental exposures during pregnancy, or genetic factors that may have contributed to preterm birth risk or risk of acute healthcare utilization after NICU discharge. These factors may affect the risk of readmission in infants with BPD and may play an even larger role than ambient air pollution, especially for infants who may not leave home very often. In the absence of information on more granular household or indoor air pollution exposures, the EJI and the air pollutants included in the study may serve as proxies for other adverse exposures, also making it difficult to dissect the true relative impact of each exposure.

In conclusion, we found that environmental determinants of health were associated with increased acute respiratory illness among infants with BPD, even after controlling for clinical characteristics, demographics, and neighborhood deprivation. Diesel particulate matter and cancer-causing pollutants may contribute to differences in healthcare utilization between non-Hispanic Black and non-Hispanic White infants with BPD. Future studies should investigate the contribution of other common air pollutants, such as PM_10_ and NO_2_, to racial disparities in outcomes of preterm infants with BPD. A deeper understanding of environmental factors that contribute to disparities in adverse outcomes in infants with BPD will allow families and policymakers to craft strategies to optimize health outcomes and equity.

## Figures and Tables

**Table 1:** Bivariable associations of infant characteristics and environmental exposures with medically attended acute respiratory illness (ED visits or hospital readmissions).

Characteristics	Overall (*n* = 378)	No Medically Attended Acute Respiratory Illness (*n* = 203, 54%)	Medically Attended Acute Respiratory Illness (*n* = 175, 46%)	*p* ^a^
EJI, Mean (SD)	0.69 (0.28)	0.65 (0.28)	0.74 (0.27)	0.002
SVM, Mean (SD)	0.58 (0.31)	0.53 (0.31)	0.64 (0.30)	<0.001
EBM, Mean (SD)	0.79 (0.16)	0.78 (0.16)	0.80 (0.16)	0.259
HVM, Mean (SD)	0.43 (0.34)	0.38 (0.34)	0.48 (0.34)	0.006
Air pollution Domain, Mean (SD)	0.82 (0.15)	0.79 (0.16)	0.86 (0.12)	<0.001
Ozone, Mean (SD)	0.81 (0.08)	0.81 (0.09)	0.81 (0.07)	0.807
PM_2.5_, Mean (SD)	0.79 (0.14)	0.76 (0.16)	0.82 (0.11)	<0.001
DSLPM, Mean (SD)	0.72 (0.16)	0.68 (0.17)	0.77 (0.14)	<0.001
ATCR, Mean (SD)	0.62 (0.16)	0.58 (0.17)	0.69 (0.13)	<0.001
BDI, Mean (SD)	0.40 (0.16)	0.37 (0.15)	0.44 (0.16)	<0.001
GA (weeks), Median [IQR]	27 [25–29]	26 [24–28]	27 [26–29]	0.641
Birth weight (g), Median [IQR]	774 [577–971]	794 [606–982]	770 [555–986]	0.662
Female Sex, *n* (%)	158 (42)	84 (41)	74 (42)	0.941
Multiple Gestation, *n* (%)	91 (24)	45 (22)	46 (26)	0.416
BPD Severity Grade ^b^, *n* (%)				<0.001
Grade 1	127 (34)	85 (42)	42 (24)	
Grade 2	153 (40)	80 (39)	73 (42)	
Grade 3	98 (26)	38 (19)	60 (34)	
Infant Race/Ethnicity, *n* (%)				<0.001
Non-Hispanic Black	189 (50)	79 (39)	110 (63)	
Non-Hispanic White	99 (26)	67 (33)	32 (18)	
Other	90 (24)	57 (28)	33 (19)	
Insurance, *n* (%)				0.064
Private	143 (38)	86 (42)	57 (33)	
Public	235 (62)	117 (58)	118 (67)	

Missing data: birth weight (*n* = 7). ED, emergency department; BPD, bronchopulmonary dysplasia; EJI, Environmental Justice Index; SVM, Social Vulnerability Module; EBM, Environmental Burden Module; HVM, Health Vulnerability Module; DSLPM, Diesel Particulate Matter; ATCR, Air Toxic Cancer Risk; BDI, Brokamp Deprivation Index; GA, gestational age, ^a^ Two-way *t* test and Wilcoxon rank sum analysis used for continuous variables. Chi-square analysis used for categorical variables. ^b^ BPD severity stratified using Jensen criteria [15].

**Table 2:** Adjusted odds of medically attended acute respiratory illness, ED visits, and inpatient readmissions per standard deviation increment increase in CDC Environmental Justice Index variables (*n* = 378).

Outcome	Composite Medically Attended Acute Respiratory Illness		ED Visits without Inpatient Readmission		Inpatient Readmissions	
Indicator	aOR	(95% CI)	aOR	(95% CI)	aOR	(95% CI)
Environmental Justice Index ^a^						
Unadjusted	1.38	(1.12–1.69)	1.76	(1.21–2.55)	1.28	(1.02–1.59)
Adjusted	1.39	(1.10–1.76)	1.61	(1.08–2.39)	1.31	(1.03–1.70)
Environmental Burden Module						
Unadjusted	1.15	(0.90–1.45)	1.49	(0.97–2.27)	1.06	(0.82–1.36)
Adjusted	0.98	(0.76–1.27)	1.20	(0.76–1.88)	0.93	(0.70–1.22)
Air Pollution Domain						
Unadjusted	1.94	(1.44–2.61)	2.73	(1.47–5.12)	1.78	(1.30–2.42)
Adjusted	1.58	(1.16–2.15)	1.78	(0.96–3.32)	1.53	(1.10–2.13)
Ozone Indicator						
Unadjusted	1.03	(0.80–1.34)	1.07	(0.70–1.61)	1.02	(0.77–1.35)
Adjusted	1.09	(0.83–1.44)	1.14	(0.69–1.86)	1.08	(0.80–1.45)
PM_2.5_ Indicator						
Unadjusted	1.72	(1.27–2.32)	2.18	(1.17–4.03)	1.61	(1.17–2.22)
Adjusted	1.43	(1.05–1.94)	1.57	(0.84–2.92)	1.39	(1.00–1.94)
DSLPM Indicator						
Unadjusted	2.06	(1.57–2.71)	2.45	(1.52–3.95)	1.95	(1.46–2.62)
Adjusted	1.78	(1.31–2.43)	1.75	(1.02–2.99)	1.78	(1.28–2.50)
ATCR Indicator						
Unadjusted	2.10	(1.59–2.78)	2.77	(1.68–4.56)	1.93	(1.43–2.61)
Adjusted	1.71	(1.24–2.35)	1.86	(1.07–3.23)	1.66	(1.18–2.34)

ED, emergency department; CDC, Centers for Disease Control and Prevention; OR, odds ratio; CI, confidence interval; EJI, Environmental Justice Index; DSLPM, Diesel Particulate Matter; ATCR, Air Toxic Cancer Risk. ^a^ Models adjusted for gestational age, sex, birth year, BPD grade, insurance, neighborhood deprivation; except for EJI, which was only adjusted for GA, sex, birth year, BPD grade, and insurance because EJI has another vulnerability indicator.

**Table 3:** Mediation * of the non-Hispanic Black and non-Hispanic White disparity in medically attended acute respiratory illness (overall, composite outcome); ED visits; and inpatient readmission per standard deviation increment higher CDC Environmental Justice Index variable in Philadelphia BPD patient cohort (*n* = 378).

	Composite Medically Attended Acute Respiratory Illness		ED Visits without Inpatient Readmission		Inpatient Readmissions	
Indicator	Mediation	*p*-Value	Mediation	*p*-Value	Mediation	*p*-Value
Environmental Justice Index	−56%	0.338	−57%	0.346	−66%	0.354
Environmental Burden Module	−0.6%	0.950	−0.5%	0.970	−0.5%	0.920
Air Pollution Domain	28%	0.050	27%	0.050	27%	0.030
Ozone Indicator	−0.2%	0.880	−0.2%	0.864	−0.2%	0.900
PM_2.5_ Indicator	16%	0.090	17%	0.124	16%	0.100
DSLPM Indicator	39%	0.004	40%	0.008	40%	0.002
ATCR Indicator	34%	0.006	34%	0.020	35%	0.012

ED, emergency department; CDC, Centers for Disease Control and Prevention; DSLPM, Diesel Particulate Matter; ATCR, Air Toxic Cancer Risk. * Model adjusted for gestational age, infant sex, birth year, BPD grade, insurance status, and neighborhood deprivation.

## Data Availability

The datasets generated during/or analyzed during the current study and the code used to analyze and manage the data are available from the corresponding author on reasonable request.

## Supplementary Materials

The following supporting information can be downloaded at: https://www.mdpi.com/article/10.3390/ijerph21050648/s1, Table S1: Adjusted^a^ odds of medically attended acute respiratory illness (overall, composite outcome); Table S2: CDC Environmental Justice Index Indicators [16].
