# Supplementary material for: Environmental Determinants of Post-Discharge Acute Respiratory Illness among Preterm Infants with Bronchopulmonary Dysplasia
Source: Int J Environ Res Public Health. 2024 May 20;21(5):648. doi: 10.3390/ijerph21050648 (PMC11120865; doi:10.3390/ijerph21050648)
Supplement: Supplementary file 1 [file ijerph-21-00648-s001.zip › ijerph-2997734-supplementary.pdf]

Table S1. Adjusted[a] odds of medically attended acute respiratory illness (overall, composite outcome); ED visits; and inpatient readmission per standard deviation increment higher CDC Environmental Justice Index variables in Philadelphia BPD patient cohort (n = 378) with the addition of race and ethnicity.

**Outcome**	**Composite Medically Attended Acute Respiratory Illness**		**ED visits without inpatient readmission**		**Inpatient readmissions**	
**Indicator**	**aOR**	**(95% CI)**	**aOR**	**(95% CI)**	**aOR**	**(95% CI)**
**Environmental Burden Module**	0.98	(0.76-1.28)	1.19	(0.76-1.88)	0.93	(0.71-1.23)
**Air Pollution Domain**	1.50	(1.10-2.06)	1.72	(0.92-3.22)	1.45	(1.04-2.03)
**Ozone Indicator**	1.07	(0.81-1.42)	1.12	(0.68-1.86)	1.05	(0.78-1.43)
**$PM_{2.5}$ Indicator**	1.34	(0.98-1.84)	1.50	(0.80-2.83)	1.30	(0.93-1.83)
**DSLPM Indicator**	1.72	(1.25-2.36)	1.69	(0.98-2.91)	1.73	(1.22-2.44)
**ATCR Indicator**	1.62	(1.17-2.24)	1.79	(1.02-3.13)	1.57	(1.11-2.23)

ED, emergency department; *CDC*, Centers for Disease Control and Prevention; *GA*, gestational age; *BPD*, bronchopulmonary dysplasia; OR, odds ratio; *CI*, confidence interval; *DSLPM*, Diesel Particulate; Matter; *ATCR*, Air Toxic Cancer Risk.

[a] Adjusted for GA, sex, birth year, BPD grade, insurance, race and ethnicity, neighborhood deprivation.

Table S2. CDC Environmental Justice Index Indicators [16].

Social Vulnerability	Racial/Ethnic Minority Status	Minority Status
	Socioeconomic Status	Poverty
		No High School Diploma
		Unemployment
		Housing Tenure
		Housing Burdened Lower-Income Households
		Lack of Health Insurance
		Lack of Broadband Access
	Household Characteristics	Age 65 and Older
		Age 17 and Younger
		Civilian with a Disability
		Speaks English “Less than Well”
	Housing Type	Group Quarters
		Mobile Homes
Environmental Burden	Air Pollution	Ozone
		PM2.5
		Diesel Particulate Matter
		Air Toxics Cancer Risk
	Potentially Hazardous & Toxic Sites	National Priority List Sites
		Toxic Release Inventory Sites
		Treatment, Storage, and Disposal Sites
		Risk Management Plan Sites
		Coal Mines
		Lead Mines
	Built Environment	Recreational Parks
		Houses Built Pre-1980
		Walkability
	Transportation Infrastructure	High-Volume Roads
		Railways
		Airports
	Water Pollution	Impaired Surface Water
Health Vulnerability	Pre-existing Chronic Diseases Burden	Asthma
		Cancer
		Higher Blood Pressure
		Diabetes
		Poor Mental Health
